# *Micropublication*: incentivizing community curation and placing unpublished data into the public domain

**DOI:** 10.1093/database/bay013

**Published:** 2018-03-02

**Authors:** Daniela Raciti, Karen Yook, Todd W Harris, Tim Schedl, Paul W Sternberg

**Affiliations:** 1 Division of Biology and Biological Engineering 156-29, California Institute of Technology, Pasadena, CA 91125, USA; 2 Informatics and Bio-computing Platform, Ontario Institute for Cancer Research, Toronto, ON M5G0A3, Canada; 3 Department of Genetics, Washington University School of Medicine, St Louis, MO 63110, USA

## Abstract

Large volumes of data generated by research laboratories coupled with the required effort and cost of curation present a significant barrier to inclusion of these data in authoritative community databases. Further, many publicly funded experimental observations remain invisible to curation simply because they are never published: results often do not fit within the scope of a standard publication; trainee-generated data are forgotten when the experimenter (e.g. student, post-doc) leaves the lab; results are omitted from science narratives due to publication bias where certain results are considered irrelevant for the publication. While authors are in the best position to curate their own data, they face a steep learning curve to ensure that appropriate referential tags, metadata, and ontologies are applied correctly to their observations, a task sometimes considered beyond the scope of their research and other numerous responsibilities. Getting researchers to adopt a new system of data reporting and curation requires a fundamental change in behavior among all members of the research community. To solve these challenges, we have created a novel scholarly communication platform that captures data from researchers and directly delivers them to information resources via *Micropublication*. This platform incentivizes authors to publish their unpublished observations along with associated metadata by providing a deliberately fast and lightweight but still peer-reviewed process that results in a citable publication. Our long-term goal is to develop a data ecosystem that improves reproducibility and accountability of publicly funded research and in turn accelerates both basic and translational discovery.

**Database URL**: www.micropublication.org

## Introduction 

Many publicly funded research results are not shared with the public, or easily findable, for a number of reasons. First, data remain unpublished due to space limitations imposed by science journals; project limitations due to funding, omission in narratives due to publication bias, e.g. negative results suffering from the file drawer effect, results that are not groundbreaking are not published in favor of research that is exciting and reports positive findings (1). Second, many data are hidden behind journal paywalls. Third, databases that help disseminate data to the public in useful scientific context with related data do not have the resources to curate at the rate of current literature growth.

To capture these ‘orphan’ or hidden data and make it easy for researchers to share early research findings through databases, we have launched a platform—*Micropublication*, with the first public facet, *Micropublication: biology*—designed to allow researchers to directly submit their data while publishing them as micropublications, according to findable, accessible, interoperable and reproducible (FAIR) data principles (2).

Publications containing a minimal unit of data have been proposed in the past (3–6). Nanopublications have been defined as the smallest unit of assertion with a semantic relationship such as an RDF(Resource Description Framework) triple of subject-verb-predicate. Nanopublications exist in the semantic web http://npmonitor.inn.ac, and serve an important purpose of being computable; however they are not human readable nor easily interpretable without programming ability. The micropublication has been more loosely defined, to contain the same semantic component with assertion as nanopublications (3), but with some leeway to contain more explanatory free text. Interest in adopting micropublications into the biomedical field has proven difficult. PLOS Currents (http://currents.plos.org/) from the publishers of PLOS journals, aimed at the rapid dissemination of research through the publication of shorter than normal articles. However, even though the articles were short, they did not adhere to any semantic constructions.

Model Organism Databases (MODs), such as WormBase, Flybase and ZFIN (Zebrafish Genome Database), have invested much effort into supplying routes for community participation in the curation pipeline. These take the form of providing an easy way for the author to communicate to the database about the data in their paper, having them triage their paper for curation, or by providing submission forms or templates to authors so they can submit data or datasets to the database directly (7–9). PomBase, the authoritative database for *Schizosaccharomyces pombe*, has successfully developed a community curation portal, Canto, that provides an intuitive curation interface for both curators and researchers, to support community curation of gene ontology (GO) terms, phenotypes, interactions and protein modifications (10). All these databases reach out to their community to seek their participation and it is successful to a fair degree, however, there still remains quite a bit of backlog.

The utility and efficiency of our novel *Micropublication* pipeline relies on creating data submission interfaces that guide authors to use community-defined standard vocabulary and ontologies by providing autocomplete fields, and dropdown lists, essential for subsequent parsing of results into databases. Upon submission, each micropublication is sent for peer-review, which is essential both for those who generate the data and for those who will consume the validated and accepted data. If accepted, the article is assigned a Digital Object Identifier (DOI), providing authors with a citable publication. In this way, the system directly trains authors to curate their own data; allows databases to capture data that do not fit into the standard narrative format of published research; enhances the efficiency of curation at these databases; and rewards authors for their work. In our model, individual findings of high scientific standard are disseminated to the community through their integration with prior knowledge by semi-automated incorporation into authoritative databases as metadata, where it can be mined through the existing interface. By streamlining this integration of data, content from each submission is automatically annotated and placed in context with relevant existing objects in the information resource (e.g. MODs and beyond), which have been actively extracted and curated from the literature for almost two decades. Data dissemination occurs through both existing information resources and the *Micropublication: biology* website.

This approach meets the top-level guidelines of the Joint Declaration of Data Citation Principles, arrived at through the collective efforts of members from a wide range of scholarly organizations and endorsed by over 100 groups invested in scholarly communication, including the National Information Standards Organization Data (11, 12). Specifically, this approach will ensure that individual results of experiments are treated the same way as other scholarly data going through traditional publication routes.

## Inaccessible data

### Curation is a non-sustainable endeavor

Authoritative public databases provide free access to data produced by researchers in their community. Curators translate published data into standardized nomenclature relevant to each biomedical field, which is critical for research in one field to be comparable with research in other fields and makes that data easily accessible for human biomedical research and discovery. Although the value of curation is immeasurable, it can be a time-consuming process and one which does not scale to the scope of modern research. Moreover, since it occurs after the course of routine research it is oftentimes considered an expensive afterthought to that research. Thus, services or tools that can ease the process of identification, extraction, translation and database deposition of data appeals to a wide range of stakeholders.

### Barriers to publication

A central expectation of taxpayer funded biomedical research is that there is a return on this investment, minimally the public dissemination of the results of research. In recent years efforts have been made to help authors comply with data dissemination requirements by creating repositories that allow the publishing of stand-alone datasets in journals Scientific Data (Nature Publishing Group), Open Data (Elsevier) and file sharing platforms (e.g. figshare and Dryad). Despite these efforts to encourage authors to share and deposit their data, there still remains an enormous gap towards compliance with these established funding and journal policies. The threshold of activation energy to submit these data into the appropriate repositories needs to be decreased and better incentivized. Moreover, article processing charges per submission or data publishing charge for data storage in data sharing platforms can heavily impact the funding resources of a laboratory for multiple submissions to these storage centers. Decreasing such costs of data deposition must be addressed.

Some effort has been made to address these limitations of the standard publication protocol. For example, the Elsevier Open data model allows researchers to deposit additional raw data as a supplementary file to be published alongside their article on ScienceDirect with an associated publishing charge. However, associated metadata is not integrated into appropriate authoritative databases and the data remain siloed at a single publisher, as is the paper until core databases are made aware of the data during curation of the primary publication. Note that if data are deposited without a reference primary research article, core databases will not know about them unless someone actively points them out. The same is true for preprint servers. While preprints solve the issue of allowing researchers to share their results with the community regardless of editorial interest, they are not peer-reviewed and so those data are not incorporated into pertinent information resources by standard biocuration processes. There is a clear and present need for authors to easily and economically comply with evolving data deposition requirements and for them to feel this effort is worthwhile. *Micropublication* is a compelling way to solve these problems in a rapid and convenient way, a platform that does not place an overly undue obstacle in the most important work of a scientist: doing experiments.

### Data/findings not reported

A significant amount of data produced by laboratories never even reach the scientific community. In published research articles, authors often refer to unpublished results but still use these data as part of the study. For example, a keyword search for ‘unpublished’ in the Textpresso corpus of *C. elegans* research, as of 2016, containing primary data of 16 500 papers identifies 5546 papers (34%) published by 540 different journals (13). The top four journals that refer to unpublished datasets are Genetics, Development, Developmental Biology and Cell with 453 (8%), 392 (7%), 300 (5.4%) and 201 (3.6%) papers, respectively, representing the top high impact journals that publish most *C. elegans* research. This situation is prevalent across all biomedical research, particularly when looking at model organism communities with larger literature corpora, for instance, a keyword search for ‘unpublished’ among mouse publications retrieves 2147/17043 (13%) documents citing unpublished results in 2010 alone. While we do not know the percentage of these excluded results that eventually got published, being able to include all supporting experimental evidence in the original submission is ideal.

In other cases, authors have data that support the study but cannot fit into the manuscript and are simply referred to as ‘data not shown’: one half of *C. elegans* papers refer to data that are not shared with the community. Finally, there are various reasons that single or a small collection of results never make it to publication, by and large because they do not fit into the longer narrative of a typical publication. One example is preliminary findings from a study that was discontinued, because the results were negative, the results indicated that the project was going in a direction peripheral to the interests of the laboratory or often because the researcher left the laboratory. Another example is an undergraduate research project or graduate student rotation project that leads to a solid, reproducible finding addressing the question of interest, but there is no publication outlet to present the single result. These circumstances lead to the data being shelved in laboratory notebooks, local computer files or lost altogether in personnel transitions typical to academic settings. A resulting consequence is that other researchers spend effort and funds unknowingly ‘rediscovering’ the finding. Not only are these cases a failure of the expectation of publicly funded research, they also can lead to a loss of knowledge within a given laboratory.

## Micropublication

Over the past decade NIH has moved to mandate researchers submit all their data to data repositories. Journal publishers such as PLOS, Elsevier and GigaScience, have increasingly encouraged authors to comply with this mandate and data repositories such as figshare and Dryad have become familiar and established data repositories. These data storage sites play important roles in data persistence. Unfortunately, because these sites do not validate data or standardize its capture, they become another data silo where information is largely lost from the community. Moreover, there is no enforcement to make authors deposit their data. Key missing players in these efforts are the genomic information resources that form the community data centers and core referential repositories for biomedical fields, in particular, the MODs. These core information resources not only collect, validate, and annotate data curated from publications and submitted datasets, they also place the data in rich context facilitating new scientific insights. *Micropublication* addresses the challenges of inaccessible data by incentivizing data submissions, by atomizing the submission and by directly delivering data to participating repositories.

**Figure 1.**
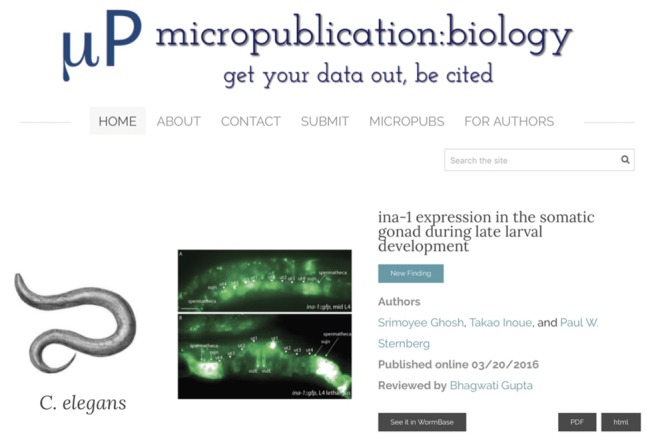
Micropublication: biology platform homepage at http://www.micropublication.org.

**Figure 2.**
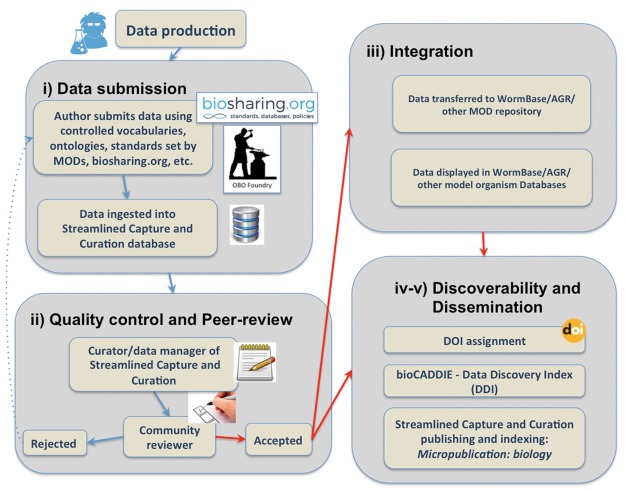
Summary of the data submission process and validation pipeline.

Micropublications incentivize researchers to place unpublished findings into the public domain. Publishing in the first facet of *Micropublication*––*Micropublication: biology––*provides a citable publication, generated in a timely fashion (Figure 1). Publishing a micropublication requires that authors populate a user-friendly web form that relies upon controlled vocabularies when available. This platform has several important and complementary results. Because submission forms are structured, they can be parsed programmatically and the contents delivered to downstream databases in a format that allows for direct incorporation. This platform also allows databases to capture data that do not fit into a narrative format of published research and automates and standardizes the capture of metadata. By relying on the expertise of authors, *Micropublication* enhances the efficiency of curation at partner databases as professional curators do not need to perform time-intensive data extraction on such submissions. More importantly, *Micropublication* has an indirect result that benefits the broader scientific community; submitting micropublications trains researchers in the value and process of deliberate, curatorial annotation and has the effect of researchers becoming more familiar with standard approaches in data sciences. The overall workflow to ensure submitted data will reach the scientific community is composed of five main components: Submission through an intuitive web interface; Content evaluation through peer-review; Integration through direct curation in WormBase; Discoverability; and Dissemination to the public through authoritative databases like WormBase and citation and indexing services. A summary of the *Micropublication* publishing process and validation pipeline is represented in Figure 2 and discussed in detail below.

### Data submission

To achieve data capture at the time of data production, we piloted an intuitive, simple submission interface with a low energy threshold for participation. This interface trains authors on the use of controlled vocabularies by providing autocomplete fields on established vocabularies and ontologies that are used by WormBase curators during normal literature curation of the standard primary research article and that meet the biosharing standards (14, 15). Each data type, such as expression patterns or mutant phenotypes, collected at a database has their own sets of vocabulary and dependencies. We allow authors to flag the submission as (i) New findings, (ii) Replication: successful, (iii) Replication: unsuccessful, (iv) Negative results and (v) Methods and Reagents. This allows submission and dissemination of novel, negative or conflicting data.

**Figure 3.**
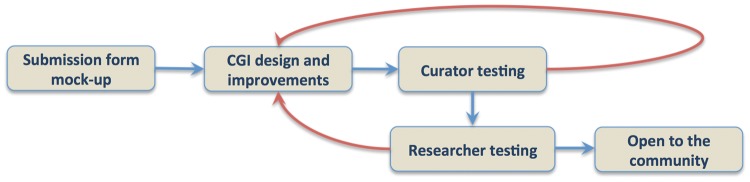
Iterative gene expression submission form design.

Submission forms design require working with curators to establish author templates that are flexible enough to adapt to whatever data type needs to be collected and establish pipelines that automate the process of author data submission and direct deposition into the database, with minimal curation oversight. As the data submission process is coupled to a publication pipeline, we are collaborating with the Collaborative Knowledge Foundation (Coko; https://coko.foundation) that offers a flexible publication management environment. Coko is developing tools that allow authors a quick, directed and intuitive interface while also triggering a number of automated alerts to the editors, reviewers and database curators that data have been submitted. These tools allow seamless communication between all stakeholders in all of these roles, while tracking the submission through the various steps required to deliver a high quality final published product and suitably formatted data for incorporation into participating data repositories. We are using iterative design and recruiting members of the science community to act in the roles of authors and reviewers to create, test and establish suitable forms and communications, adopting a User-centered Design approach (16, 17). Specifically, we created a pilot submission form for gene expression data through such an iterative process (Figure 3). We generated wireframe and later live form prototypes and then collected user feedback to improve these initial designs. We engaged in one-on-one testing and made sure that the user’s experience would be as intuitive as possible. We went through several rounds of iterative feedback from the community to gather suggestions and recommendations.

Our submission guidelines comply with the Minimum Information Standards for scientific data reporting and current standards (Minimum Information for Biological and Biomedical Investigations-Biosharing portal, biosharing.org/standards) (14, 15). For example, for gene expression results, the guidelines are modeled after the MISFISHIE specifications [Minimum information Specification For *In Situ* Hybridization and immunohistochemistry Experiments, (18)], comply with WormBase curation standards and data models and are intended to define a set of minimum information needed to interpret and reproduce an individual experiment aimed to localize the expression of a transcript or a protein. Metadata and annotations captured through this simple interface populate local postgres database tables and enter the regular WormBase data flow upon reviewers’ approval. Concomitantly, the submitted data, with an accompanying image, are converted into a publication style html and PDF documents and published on the *Micropublication: biology* website.

**Figure 4.**
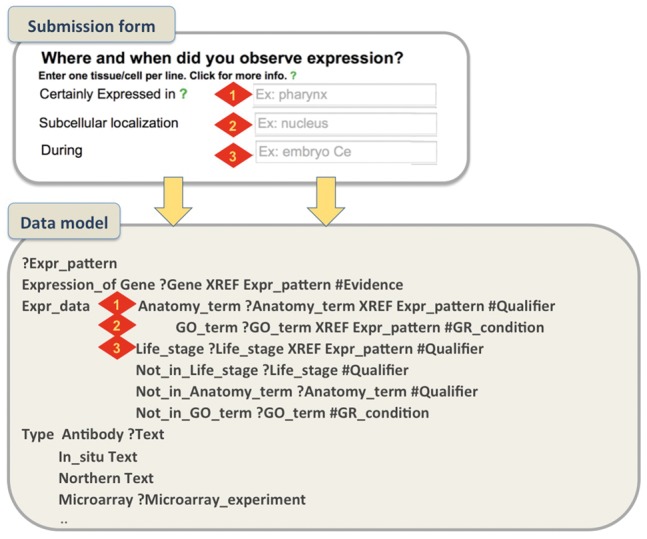
Submission form and WormBase Expression pattern data model. A simplified example that shows how metadata captured through the form represent specific data fields in the data model. For gene expression, authors can describe the spatio-temporal localization of a transcript/protein by choosing terms from pre-defined ontologies. We use the *C. elegans* anatomy ontology to describe localization in cells/tissues, the GO Cellular Component Ontology to describe subcellular localization and the *C. elegans* developmental ontology to capture temporal expression. We allow authors to choose from pre-designed qualifier fields (certainly expressed, partially expressed, possibly expressed and NOT expressed) that allow a more detailed description of the pattern.

An example on how the submission form aims to capture the metadata required in the Expression pattern model is shown in Figure 4.

### Quality control and peer-review

Data quality and completeness are overseen at multiple levels through the submission process. First, the technical quality of the submission should meet the highest standards by making sure all mandatory fields are completed. Since authors are using pre-designed forms, we can make sure that any required information is supplied at the time of submission. In addition, the forms have embedded quality checks of known entities and community standard vocabularies. For example, only known gene names can be entered. If authors are submitting data for a novel gene, the forms will start a dialog with WormBase curators to ensure that the name complies with community-approved nomenclature. In addition, all entities entered through the forms will elicit a popup box that contains known information about that entity so authors can verify each object; this function serves to avoid typos and inconsistencies in submission.

Second, submissions received through the forms are quickly scanned by a Managing Editor/Curator, as a spam check and to make sure there aren’t obvious technical problems with the submission, such as inclusion of correct figure, unlabeled figure panels, etc. The submission is then sent to a Community Science Editor for final approval to send to a reviewer. The data are scanned by the Community Science Editor for completeness and for agreement with any submitted narrative. Since these submissions are designed to be single experimental results, the submission quality control (QC) step is inherently quick.

Third, QC’d submissions are sent for peer-review to a community expert. We identify experts through author submitted suggestions and by identifying people in the community who have published data similar to what is being submitted. Since checks on quality are already partially built into the form through technical implementation, such as controlled vocabularies, autocompletion and mandatory fields, the role of the reviewer is to provide a judgment on whether the experimental evidence likely represents the stated observation. Specifically, reviewers are tasked with evaluating the scientific validity of the submission and assessing if all the pertinent information to reproduce the experiment is provided. For example if an author states that ‘Fluorescence is reported in the AIY neuron’ that neuron should be unambiguously identified by its morphological features or by colocalization with known markers. Article acceptance is a simple stamp of approval from the reviewer and does not involve evaluation of the findings in the context of a complex narrative, i.e. the sentence ‘gene A is observed to be expressed in cell B’ is a purely descriptive observation that differs from speculating the function of gene A in that specific cell, an assertion that should be corroborated by additional scientific evidence. Finally, accepted articles are processed through a text hyperlinking step (19) to identify, extract and link relevant or potential biological entities, which are used to automatically validate known entities and alert database curators to possible new entities that need to be entered into the database.

### Integration

Once the article is accepted by the reviewer and approved by the curator, it enters the WormBase––or other database, such as Model Organism (MOD) repository––data flow as any other curated object, i.e. a data file is sent from the relational database and enters the receiving database’s build process. The data are available on the pertinent web page in WormBase (or other information resource). As a result of this, data are publically shared and searchable alongside data curated from the literature.

### Discoverability

In addition to these data being available through the authoritative databases, every accepted submission is assigned a DOI, which can be used as an immediate citation. We currently use the California Digital Library (CDL) EZID services, however, we will be moving to another servicer as the CDL will soon only service University of California institutions. Soon *Micropublication* articles will be discoverable through Datamed, the new data discovery index platform developed in the context of the Big Data to Knowledge (BD2K) initiative -bioCADDIE (20). Eventually, our publications will also be indexed in established science index services such as PubMed to maximize access to, reuse of and repurpose of these research results.

### Dissemination and outreach to the scientific community

The success of this project relies heavily on proper outreach. We have already started to engage researchers of the highly collaborative *C. elegans* community. WormBase has in place different pipelines for communicating with *C. elegans* researchers in order to maximize the speed of curation and the inclusion of novel research data in this information resource. We have set up an author first pass pipeline that automatically sends an e-mail to authors as soon as their published paper is brought into our local Postgres paper corpus. We contact the authors to flag their paper for specific sets of metadata that are included, e.g. gene expression; gene regulation; mutant, RNAi, over-expression or chemical-based phenotypes; genetic interactions and so forth. We also incentivize community curation by providing authors easy-to-use forms with which they can directly curate already published results (allele-phenotype submission and concise description). Over the years, author participation in the *C. elegans* community has been between 30–40%, based on weekly outreach requesting authors to flag their papers for data pertinent to curation in WormBase.

We are continuing the dialogue with authors and are reaching out to the community by: e-mailing and calling the principal investigators to solicit submissions of already produced but unpublished data; reaching out with presentations and workshops during local and international *C. elegans* meetings; personally visiting research laboratories and engaging them in the project; using social media platforms––Twitter, Blogs––to advertise the initiative; advertising the project on the WormBase website and blog. We also initiated collaborations with key stakeholders [member and non-members of the Alliance of Genome Resource (AGR) consortium: ZFIN, FlyBase, Xenbase, SGD, MGI, RGD] to make the pipeline available to the broader scientific community.

## Preliminary results

As of 30 October 2017, of 28 *C. elegans* articles received, we have approved and published 22, rejected 2, retracted 1 on request of the author and are currently preparing 3 for publication. During these early days, we have had an average turnaround time of < 1 month (20 days), with the fastest being 2 days. We anticipate this turn-around time to get faster and more consistent as our platform is developed by Coko and communication between authors, reviewers and editors is streamlined, however, there will be lags in the process, which may not be overcome as discussed below.

**Figure 5.**
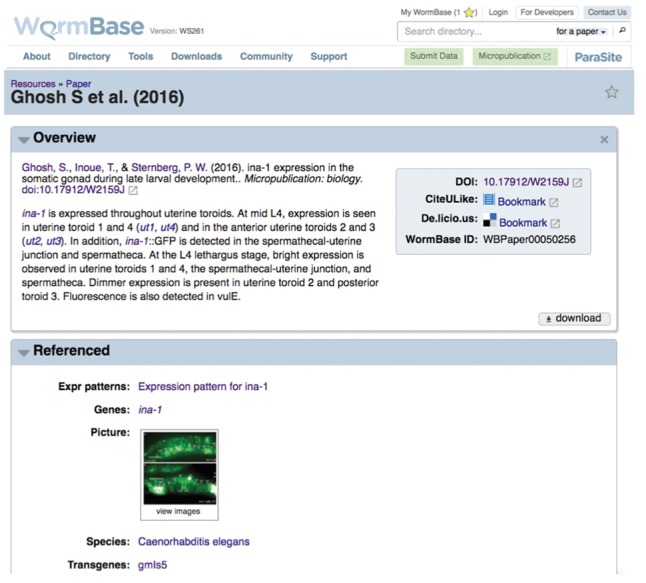
WormBase view of a gene expression micropublication available at www.wormbase.org. http://wormbase.org/resources/paper/WBPaper00050256#03–10.

The submissions we received were first sent to our Community Science Editor for compliance check and reviewer suggestions, and if approved, sent to reviewers. Managing Editors mediated the discussion between authors and reviewers. Upon acceptance, we assigned a DOI and published the articles on WormBase and on http://www.micropublicationbiology.org/. All the experimental results submitted through *Micropublication* are not only readily available on *Micropublication: biology* but are also integrated into WormBase and discoverable alongside other curated data. This essentially short circuits the normal route of data incorporation that can take months or even years to assimilate research data into public repositories. Thus, *Micropublication* is a much needed option for dissemination of biological knowledge that happens instantaneously. Authors have now become ‘biocurators,’ able to transfer their observations with the use of structured vocabularies and data are now accessible as soon as is available. A submission example integrated in WormBase for *ina-1* gene expression is discoverable (Figure 5), and is also available on the *Micropublication: biology* site http://www.micropublicationbiology.org/ghosh-et-al-2015–ina-1.html (21).

## Discussion

Curation of biomedical data is a costly and time-consuming endeavor but it is the best approach for sharing, managing, integrating and analysing existing and new data (22). While several information resources incentivize community curation in order to speed data sharing and alleviate the burden of professional curation, many authors are still unresponsive due to the lack of metrics that recognize the researcher’s contributions. In addition, bench scientists often lack the archival and curation expertise necessary for proper data integration and reuse. Our solution to these challenges is *Micropublication: biology*, which reports findings of high scientific standard to the community via online publication and automatic integration into authoritative databases (i.e. MODs). Such integration is attractive because data are best preserved and mined in repositories managed by trusted entities for long-term access. Findings in the journal are citable—via assignment of a DOI and ultimately a PubMed ID—and thus are readily discoverable by the research community. To ensure validity, *Micropublication* articles comply with the Minimum Information Standards for scientific data reporting and undergo streamlined peer-review by domain experts with semi-automated assignment of reviewers. The submissions interface forces the use of standard vocabularies by providing autocomplete fields, essential for subsequent parsing of results into databases.

Micropublications help to drive the standardization of curation by supplying data submission interfaces that assist in the assignment of metadata by proposing terms from accepted vocabularies and ontologies. Our tools and templates facilitate consistent use of community-defined standards such as common data elements and standards used by archival resources, e.g. MODs and other NIH supported BioMedical Databases. Overall, this improves the speed and accuracy of extracting metadata information through automated and semi-automated approaches; we link experimental results to authoritative digital repositories, allowing heterogeneous data to be harmonized and merged. This supports data annotation at the point of publication and is presented with full experimental methods in a structure that supports reproducibility and public sharing of reagents and data. Data are captured closer to the point of data generation than in typical publishing strategies, and the review step ensures that reported data are complete and adhere to community standards. Authors are incentivized to participate as *Micropublication* articles are fully citable publications. The impact of this change can be tremendous, as researchers now have means of accessing data that was shared suboptimally––or not shared at all––and makes use of these results to further accelerate the pace of scientific discovery.

This publication model bridges researchers to data repositories and literature repositories which we believe will turn *Micropublication* into a natural addition to scholarly communication. First, we intimately tie publication submission to curation in a community supported database allowing structured data capture and machine accessibility for full data dissemination as proposed (3). Second, we peer-review these research snippets, which is imperative to researchers for establishing trust in results. Third, we are involving our community in the design of the forms and the process of review.

At this stage, each new submission informs the evolution of *Micropublication*, in terms of what the community is willing to submit as well as how well each compartmentalized form for a specific data type functions. For example, an expression pattern easily fits in a semantic expression, however, a result that involves a phenotype observation requires more contextual information that often extends beyond the bounds of current controlled vocabularies. We have demonstrated a proof of principle for submission of *C. elegans* gene expression data and drug induced phenotype (http://www.micropublicationbiology.org/). The priority of incorporating additional data-types will be community-driven. For example, authors expressed interest in micropublishing results for data types for which we did not have a submission form, such as gene locus mapping data, genetic screen results and variation sequence data. For these and future requests where we do not have a ready submission form, we provide a simple word template that authors can use to submit their data. As we progress with our publication platform, we will continue to prioritize the forms’ design according to the interest of the community. We aim to test whether the *Micropublication* paradigm can be implemented cost-effectively and adopted broadly by the biological research community. In the longer term, this open science model can be extended to other disciplines. The Micropublication project is supported until 2020 by a grant from the National Institutes of Health. After the development and establishment of our platform we will be able to project what will be our maintenance and expansion costs, allowing us to explore future funding models.

### Risks and challenges

The biggest challenge in this project is to overcome researcher hesitation to share data through a new venue. There is tight competition for research funds in the biomedical field which results in a culture of fear of losing out on data provenance. This is in large part exacerbated by using the rate of publication in ‘high-impact’ journals as a metric for a researcher’s value in science. In addition, the publication industry has over the years created a limited avenue of ‘accepted’ and ‘valued’ science communication, resulting in incomplete or biased publication of data. Authors are limited in physical space in their articles keeping them from publishing all their data. In addition authors do not publish negative data, which makes up the majority of all clinical studies (1). Biomedical researchers are less likely to participate in non-standard publication models, yet this field would be helped the most by this model since sharing results and resources will significantly cut down on research time and costs. The paradigm of evaluating the worth of a researcher for tenure is actively being challenged by many groups including scholars, professional societies and organizations and even publishers. However, even with all this activity in creating new avenues of research acknowledgement and dissemination, scientists remain dubious of sharing their research in novel portals.

The other challenge we need to overcome is training researchers in curation. By leveraging our collective experience curating the scientific literature, we hope to streamline this process. For example, in our experience, we have often seen an inability or hesitancy for authors to submit data despite their obvious expertise and deep knowledge of their own results. This may be because they lack the computational expertise to format the data, or an unfamiliarity with the underlying data models used at repositories, or the dependencies and relationships between data types. Clearly, due to its complexity, direct submission must be a brokered process.

Finally, since *Micropublication* is a publishing platform we are faced with some of the same challenges other publishers have confronted. One common problem is peer-review. In our publication paradigm, however, this problem is increased as we anticipate a scale-up of articles which will require an even larger pool of reviewers to avoid reviewer fatigue. We are tackling this problem in three ways. First, in certain circumstances we have opened up the pool to senior graduate students and post-doctoral researchers that are experts in the topic of the micropublication. Besides widening the number of potential reviewers, this has an even greater benefit of exposing young expert scientists to the publication process, a very useful experience for their and the scientific community’s future. Second, we are incentivizing the reviewer task by allowing reviewers to be acknowledged. Open reviews are being adopted by a number of established science journals and we anticipate that participation as a reviewer will be added into the equation of scientific value for the scientists. By allowing reviewers to be recognized we also hope to fill the missing reviewer metric gap (23). Third, while in this initial phase of the project the Editorial team will select a reviewer when authors do not provide reviewer suggestions during submission, in the near future, we will pilot selecting reviewers via text-mining approaches based on Textpresso 3.0 that we will have in place for the AGR (http://www.alliancegenome.org/) member databases (H.M. Mueller *et al.*, under revision). Reviewers will be suggested to the Managing Editor/Curator by an automated system that will parse previous publications, recognize controlled vocabulary terms in the text (e.g. anatomy ontology, gene names, etc.) and rank authors as experts in the field.

We proved that a submission can be processed in <2 business days, but we were challenged, as are other publishers, by reviewer’s responsiveness. We normally invite 1–2 scientists to review the submission and wait 4–5 business days to allow them enough time to respond to the message. If we don’t get any response, we select alternative reviewers but this process can become a rate limiting step. Given that the micropublication manuscript is a single experiment (usually containing a single figure or table) with a streamlined text, the actual review process can be done in minutes by a reviewer that is familiar with the field. We anticipate that with reviewer’s effort recognized and with the inclusion of expert senior graduate students and post-doctoral researchers, the peer-review process will have a much faster turnaround. As the reported findings that we are requesting for review are single experiments, we currently aim for a turnaround of 10 business days from author submission to reviewer acceptance. In addition, we highlight a new metric for community participation with open reviewer acknowledgement. To date, while only one reviewer has opted for anonymity, all remaining reviewers have welcomed the option of open acknowledgement.

We are confident we can overcome the challenges with which we are faced. We are working to overcome an author’s hesitance to contribute through incentives and community outreach. *Micropublications* preserve scientific provenance, giving citable credit, through widely established unique identifiers and universally accepted citations to the researcher at the potential earliest point in a scientific discovery. Since accepted data automatically travel to authoritative databases, researchers’ data achieve quicker integration into those databases. Community members that agree to review these publications achieve acknowledgement for their participation, creating a new metric for their role in scientific contribution. Finally, we are leveraging our own professional curatorial expertise to build a user-friendly experience that mitigates barriers to participation.

## References

[1] Hopewell S., Loudon K., Clarke M.J. (2009) Publication bias in clinical trials due to statistical significance or direction of trial results. Cochrane Database of Systematic Reviews, Issue 1. Art. No.: MR000006. 10.1002/14651858.MR000006.pub3 PMC8276556 19160345

[2] Wilkinson M.D., Dumontier M., Aalbersberg I.J. (2016) The FAIR Guiding Principles for scientific data management and stewardship. Sci. Data, 3, 160018. 26978244 10.1038/sdata.2016.18 PMC4792175

[3] Clark T., Ciccarese P.N., Goble C.A. (2014) Micropublications: a semantic model for claims, evidence, arguments and annotations in biomedical communications. J. Biomed. Semantics, 5, 28. 26261718 10.1186/2041-1480-5-28 PMC4530550

[4] Do L., Mobley W. (2015) Single Figure Publications: towards a novel alternative format for scholarly communication. F1000Res, 4, 268. 27506233 10.12688/f1000research.6742.1 PMC4786890

[5] Mina E., Thompson M., Kaliyaperumal R. (2015) Nanopublications for exposing experimental data in the life-sciences: a Huntington’s Disease case study. J. Biomed. Semantics, 6, 5. 26464783 10.1186/2041-1480-6-5 PMC4603842

[6] McCusker J.P., Lebo T., Krauthammer M. (2013) Next generation cancer data discovery, access, and integration using prizms and nanopublications. Data Integr. Life Sci., 7970, 105–112. 27631029 10.1007/978-3-642-39437-9_9 PMC5019567

[7] Lee R.Y.N., Howe K.L., Harris T.W. (2017) WormBase 2017: molting into a new stage. *Nucleic Acids Res.*, 45, D869–D874. 10.1093/nar/gkx998 PMC5753391 29069413

[8] Gramates L.S., Marygold S.J., Santos G.D. (2016) FlyBase at 25: looking to the future. *Nucleic Acids Res*, 45, D663–D671. 10.1093/nar/gkw1016 PMC5210523 27799470

[9] Howe D.G., Bradford Y.M., Eagle A. (2016) A scientist’s guide for submitting data to ZFIN. Methods Cell Biol., 135, 451–481. 27443940 10.1016/bs.mcb.2016.04.010 PMC6319372

[10] Rutherford K.M., Harris M.A., Lock A. (2014) Canto: an online tool for community literature curation. Bioinformatics, 30, 1791–1792. 24574118 10.1093/bioinformatics/btu103 PMC4058955

[11] Data Citation Synthesis Group: Joint Declaration of Data Citation Principles. Martone M. (ed). San Diego CA: FORCE11; 2014. 10.25490/a97f-egyk.

[12] Starr J., Castro E., Crosas M. (2015) Achieving human and machine accessibility of cited data in scholarly publications. PeerJ. Comput. Sci.1, pii: e1. 10.7717/peerj-cs.1 PMC4498574 26167542

[13] Müller H.M., Kenny E.E., Sternberg P.W. (2004) Textpresso: an ontology-based information retrieval and extraction system for biological literature. PLoS Biol, 2, e309. 15383839 10.1371/journal.pbio.0020309 PMC517822

[14] Taylor C.F., Field D., Sansone S.A. (2008) Promoting coherent minimum reporting guidelines for biological and biomedical investigations: the MIBBI project. Nat. Biotechnol., 26, 889–896. 18688244 10.1038/nbt.1411 PMC2771753

[15] McQuilton P., Gonzalez-Beltran A., Rocca-Serra P. (2016) BioSharing: curated and crowd-sourced metadata standards, databases and data policies in the life sciences. Database (Oxford). 10.1093/database/baw075 PMC4869797 27189610

[16] Pavelin K., Cham J.A., de Matos P. (2012) Bioinformatics meets user-centred design: a perspective. PLoS Comput. Biol., 8, e1002554. 22807660 10.1371/journal.pcbi.1002554 PMC3395592

[17] de Matos P., Cham J.A., Cao H. (2013) The Enzyme Portal: a case study in applying user-centred design methods in bioinformatics. BMC Bioinformatics, 14, 103. 23514033 10.1186/1471-2105-14-103 PMC3623738

[18] Deutsch E.W., Ball C.A., Berman J.J. (2008) Minimum information specification for in situ hybridization and immunohistochemistry experiments (MISFISHIE). Nat. Biotechnol., 26, 305–312. 18327244 10.1038/nbt1391 PMC4367930

[19] Rangarajan A., Schedl T., Yook K. (2011) Toward an interactive article: integrating journals and biological databases. BMC Bioinformatics, 12, 175. 21595960 10.1186/1471-2105-12-175 PMC3213741

[20] Ohno-Machado L., Alter G., Fore I. (2015) bioCADDIE white paper–Data Discovery Index. figshare.

[21] Ghosh S., Inoue T., Sternberg P. (2015) ina-1 expression in the somatic gonad during late larval development. Micropublication:biology. *Dataset*, doi: 10.17912/W2159J. 10.17912/W2159J PMC7255860 32550338

[22] Karp P.D. (2016) How much does curation cost? Database, 2016, 1–2. 10.1093/database/baw110 PMC4976296 27504008

[23] Cantor M., Gero S. (2015) The missing metric: quantifying contributions of reviewers. R. Soc. Open Sci., 2, 140540. 26064609 10.1098/rsos.140540 PMC4448813

